# Supplementary material for: Membrane cholesterol mediates the cellular effects of monolayer graphene substrates
Source: Nat Commun. 2018 Feb 23;9:796. doi: 10.1038/s41467-018-03185-0 (PMC5824811; doi:10.1038/s41467-018-03185-0)
Supplement: Supplementary file 1 — Supplementary Information [file 41467_2018_3185_MOESM1_ESM.docx]

**Membrane cholesterol mediates the cellular effects of monolayer graphene substrates**

Kitko et al.

**Supplementary Figures**

**
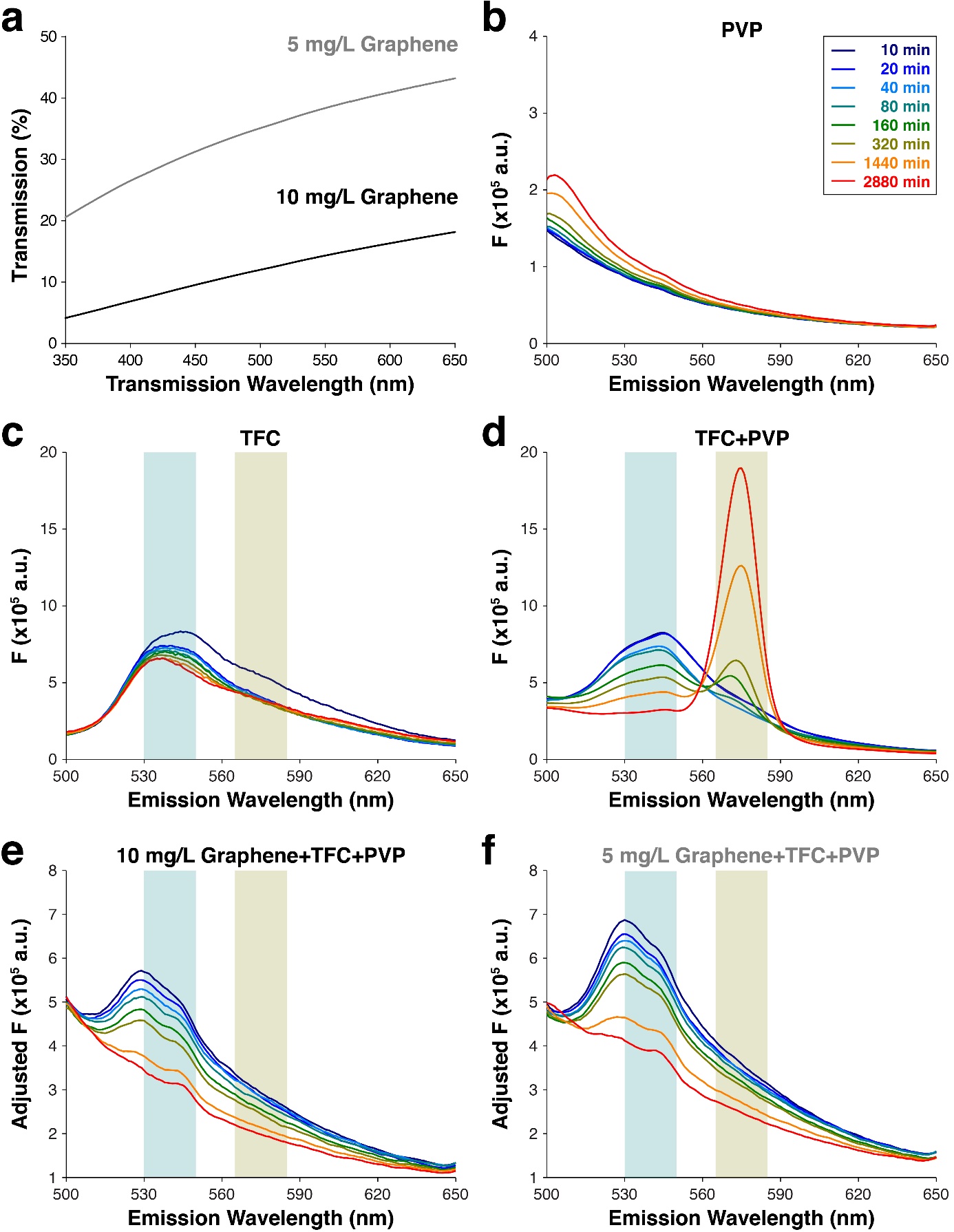
**

**Supplementary Figure 1. Spectrofluorometry demonstrates an interaction between TFC and graphene. (a)** Transmission spectra of 5 and 10 mg/L graphene (gray and black, respectively) in H_2_O with 2 wt% PVP show a concentration-dependent broadband graphene absorbance. **(b)** Time-lapse emission spectra of 2 wt% PVP in H_2_O. **(c)** 1.3 μM TFC in H_2_O shows a time-dependent fluorescence decay in the 530-549 nm range (cyan box, the peak of TFC fluorescence in H_2_O) and the 565-584 nm range (beige box). **(d)** Addition of 2 wt% PVP to 1.3 μM TFC in H_2_O. **(e)** Time-lapse emission spectra of 1.3 μM TFC in H_2_O containing 2 wt% PVP and 10 mg/L graphene, adjusted by the absorbance of 10 mg/L graphene. **(f)** Time-lapse emission spectra of 1.3 μM TFC in H_2_O containing 2 wt% PVP and 5 mg/L graphene, adjusted by the corresponding absorbance of 5 mg/L graphene.


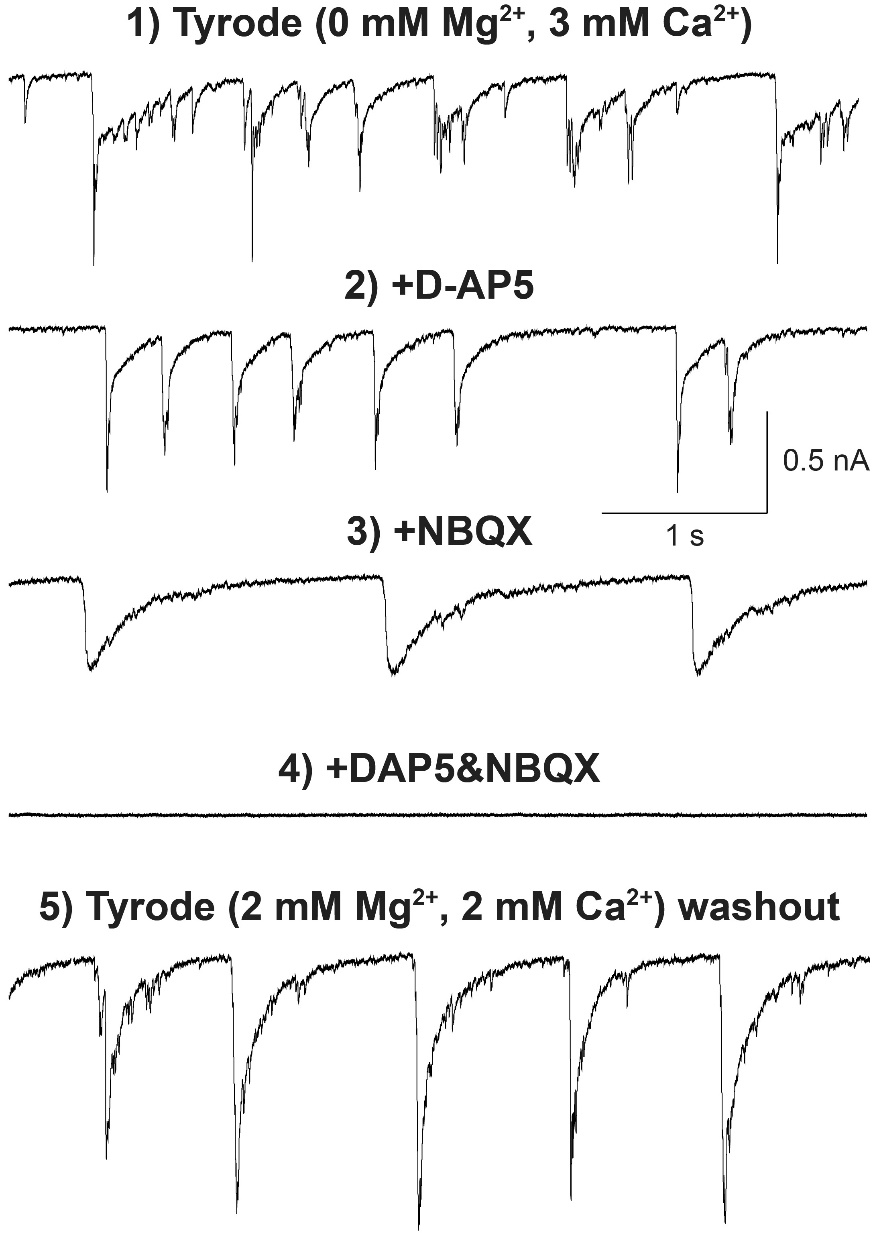


**Supplementary Figure 2. Sequential recording of AMPA and NMDA receptor-mediated EPSCs from the same neurons.** 0 mM Mg^2+^ Tyrode releases the voltage gating of NMDARs and thus results in readily distinguishable AMPAR and NMDAR contributions to the sEPSC records. Addition of the NMDAR antagonist D-AP5 isolates the fast AMPAR component. Addition of the AMPAR antagonist NBQX after wash off of D-AP5 allows the isolation of the slow NMDAR component. Addition of both D-AP5 and NBQX completely eliminates sEPSCs, and washout with 2 mM Mg^2+^ Tyrode’s solution recovers sEPSCs that are largely mediated by fast AMPAR components which can also be seen in the traces in (1). 0 mM Mg^2+^, as shown in (1), also acts to remove the NMDA receptor blockade that exists in normal physiological solution [^1^](#_ENREF_1). Thus, the contribution of NMDA-receptor mediated currents are more readily visible in (1) than in (5).

**
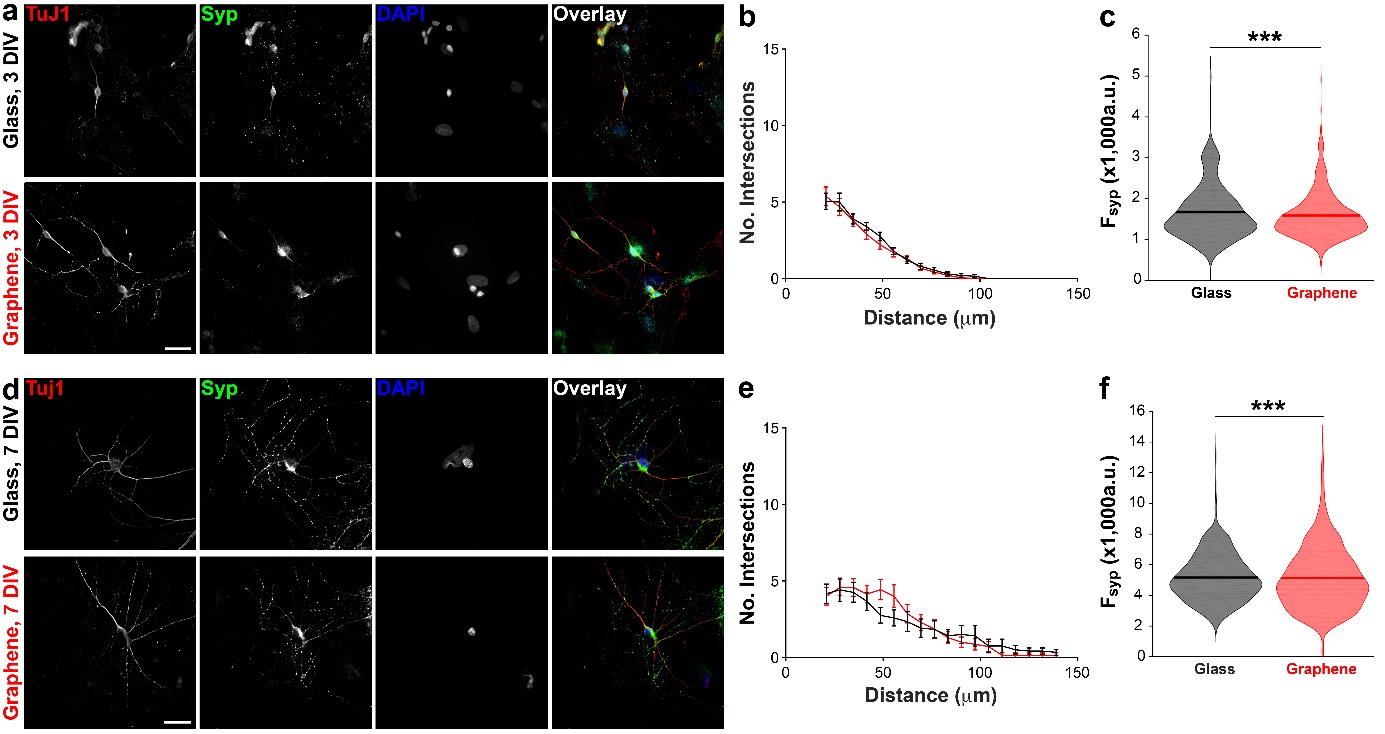
Supplementary Figure 3. Graphene does not substantially alter early synaptic development. (a)** Sample images of fluorescence immunostaining for the synaptic vesicle marker Synaptophysin (Syp, green), neuron-specific class III β-tubulin (TuJ1, red) and nuclear marker DAPI (blue) at 3 DIV. Scale bar, 50 μm. **(b)** Sholl analysis at 3 DIV (*p* > 0.05, Two-Way ANOVA with repeated measures, n_glass_ = 17 cells and n_graphene_ = 22 cells). **(c)** Intensity of Syp staining in TuJ1(+) processes at 3 DIV (n_glass_ = 1524 synapse sand n_graphene_ = 1715 synapses, *** *p* < 0.001, two-tailed *t*-test, Cohen’s *d* = 0.188) Solid lines indicate mean value. **(d)** Sample images of fluorescence immunostaining for Syp (green), TuJ1 (red) and DAPI (blue) at 7 DIV. Scale bar, 50 μm. **(e)** Sholl analysis at 7 DIV (*p* > 0.05, Two-Way ANOVA with repeated measures, n_glass_ = 17 cells and n_graphene_ = 11 cells). **(f)** Intensity of Syp staining in TuJ1(+) processes at 7 DIV (n_glass_ = 4381 synapses and n_graphene_ = 3990 synapses, *** *p <* 0.001, two-tailed *t*-test, Cohen’s *d* = 0.146). Solid lines indicate mean value. All error bars are the S.E.M. of the number of intersections from each FOV.

**
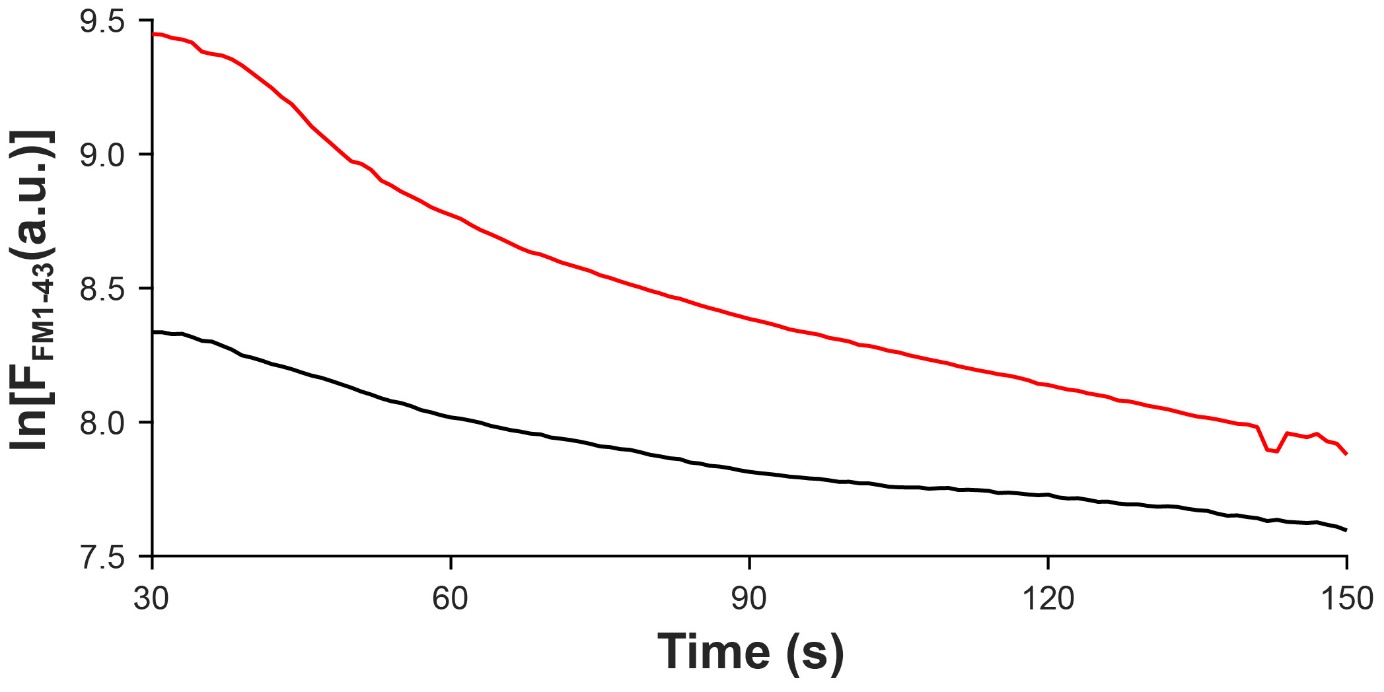
Supplementary Figure 4. FM1-43 destaining rate suggests increased vesicle release probability in neurons on graphene.** Raw FM1-43 fluorescence intensity values for graphene (red solid line) or glass (black solid line) during the stimulation period.

**
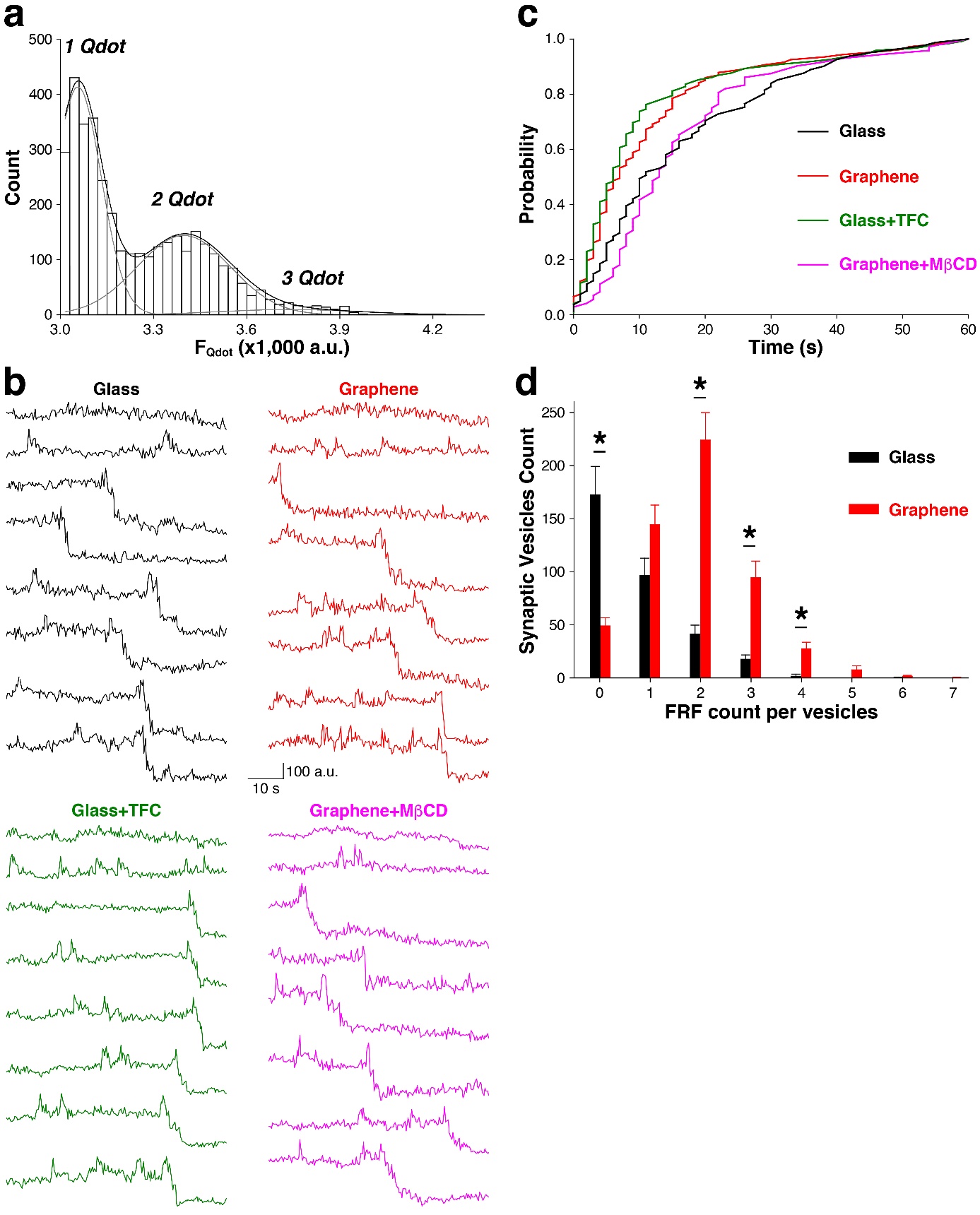
Supplementary Figure 5. Single Qdot imaging to measure single vesicle turnover. (a)** The distribution of mean intensity values (without background correction) in individual synaptic boutons defined by retrospective FM4-64 staining. The intensity distribution was fit with Gaussians of equal offset (i.e. mean and variance increase in folds). Quantal analysis (black and gray lines, Gaussian fits based on a maximal likelihood estimate) indicates that the mean intensity representing the unitary Qdot photoluminescence is 378 ± 41 a.u. with an average background intensity of 2719 ± 54 a.u. **(b)** Sample traces showing prototypical photoluminescence traces of single Qdots for the four different treatments: neurons on glass without (black) or with TFC pretreatment (green) and neurons on graphene without (red) or with MβCD pretreatment (purple). A small and transient increase in Qdot fluorescence (uptick) represents fast-and-reversible fusion (FRF), and a large and unitary decrease following an uptick represents full-collapse fusion (FCF). **(c)** Vesicle release probability (measured as the first fusion events of individual Qdot-loaded synaptic vesicles) is significantly higher in neurons on graphene (red) than on glass (black) (*p* < 0.05) but is significantly reduced by MβCD pretreatment (purple) (n_graphene_ = 231, n_graphene+MβCD_ = 214, n_glass_ = 183, n_glass+TFC_ = 225 Qdots, N = 3 for every group; *p* < 0.05, two-tailed *t*-test). In contrast, the release probability on glass (black) is significantly increased by TFC pretreatment (green) (*p* < 0.05, two-tailed *t*-test). (**d**) Number of synaptic vesicles (y-axis) conducting the specified numbers of FRF events during the stimulation (x-axis) in neurons on graphene (red) or glass (black) (n = 3 FOVs from N = 3 for each group *, *p* < 0.05, two-tailed *t*-test). Error bars are the S.E.M. from each FOV.

**
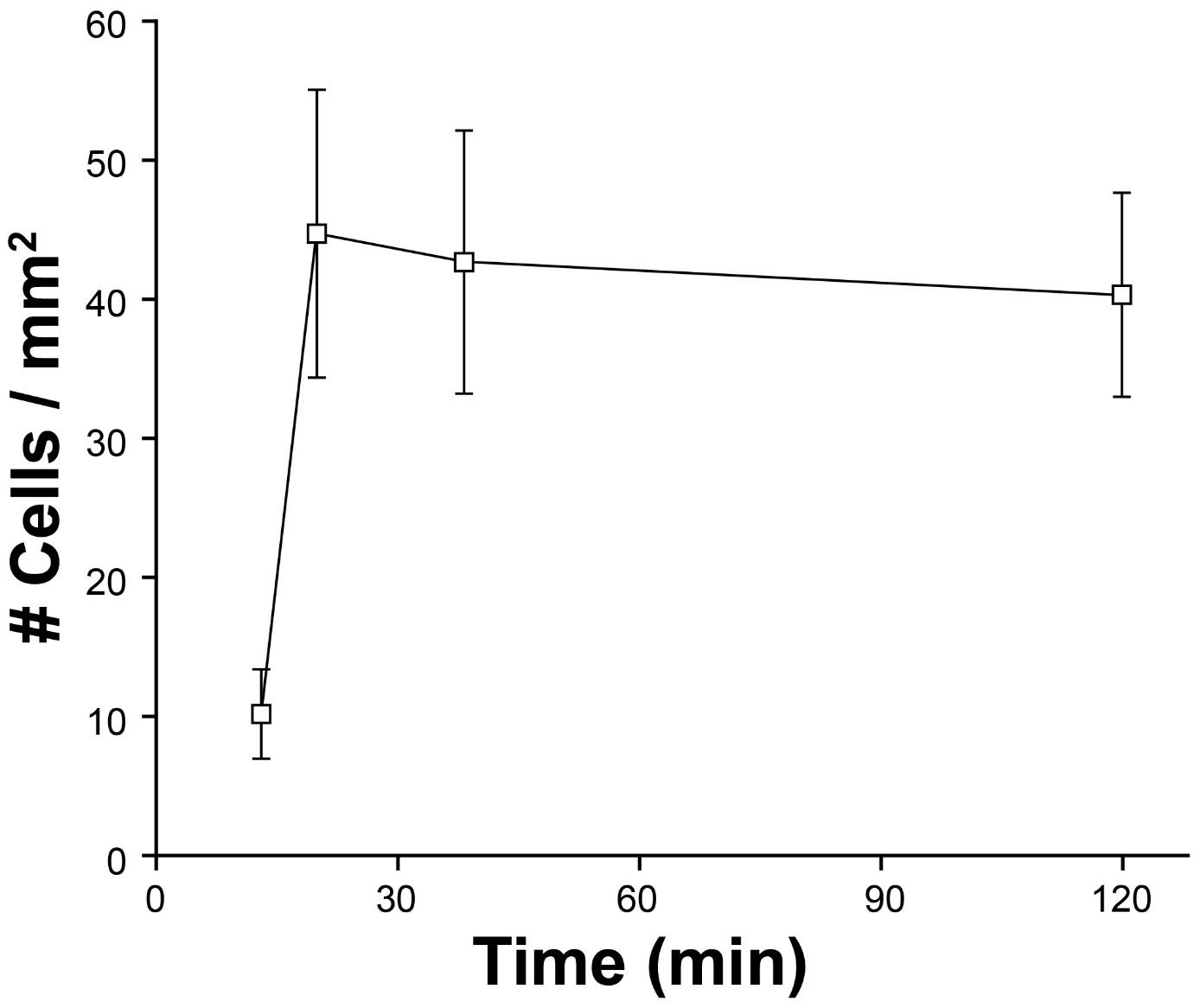
Supplementary Figure 6. Cell adhesion after plating.** Dissociated hippocampal neurons were plated on bare glass coverslips and incubated in plating media for designated periods of time before washing with Hank’s solution. Cells in randomly chosen FOVs were counted to calculate cell densities. Error bars are S.E.M for each time point.

**
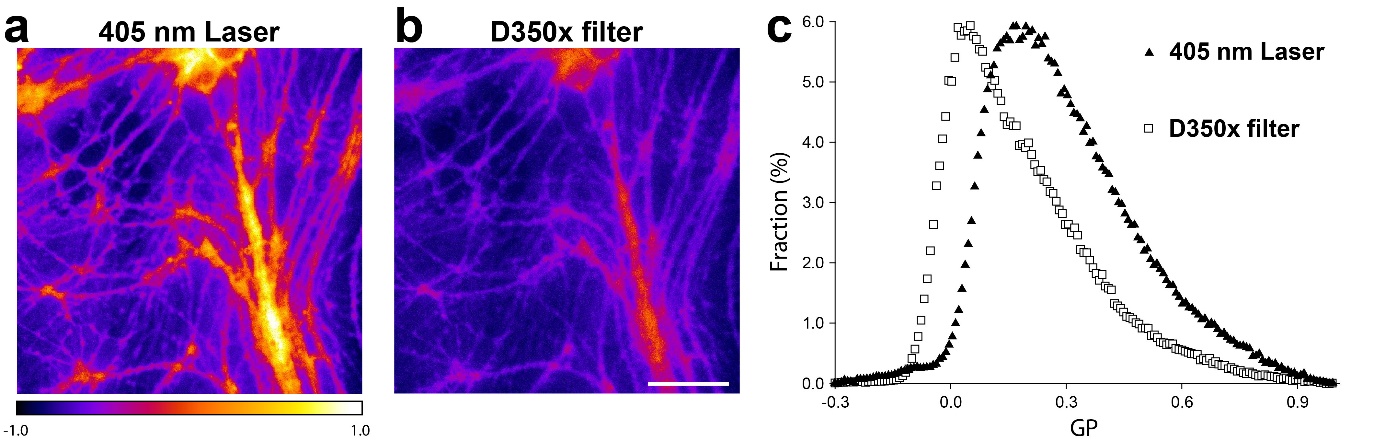
**

**Supplementary Figure 7.** **GP values with two different excitation settings.** (**a & b**) Sample GP images with a 405 nm laser (**a**) or Prior 200 light source and D350x filter (**b**)(Chroma). Scale bar, 20 μm. (**c**) The distribution of GP pixel values for both conditions.

**
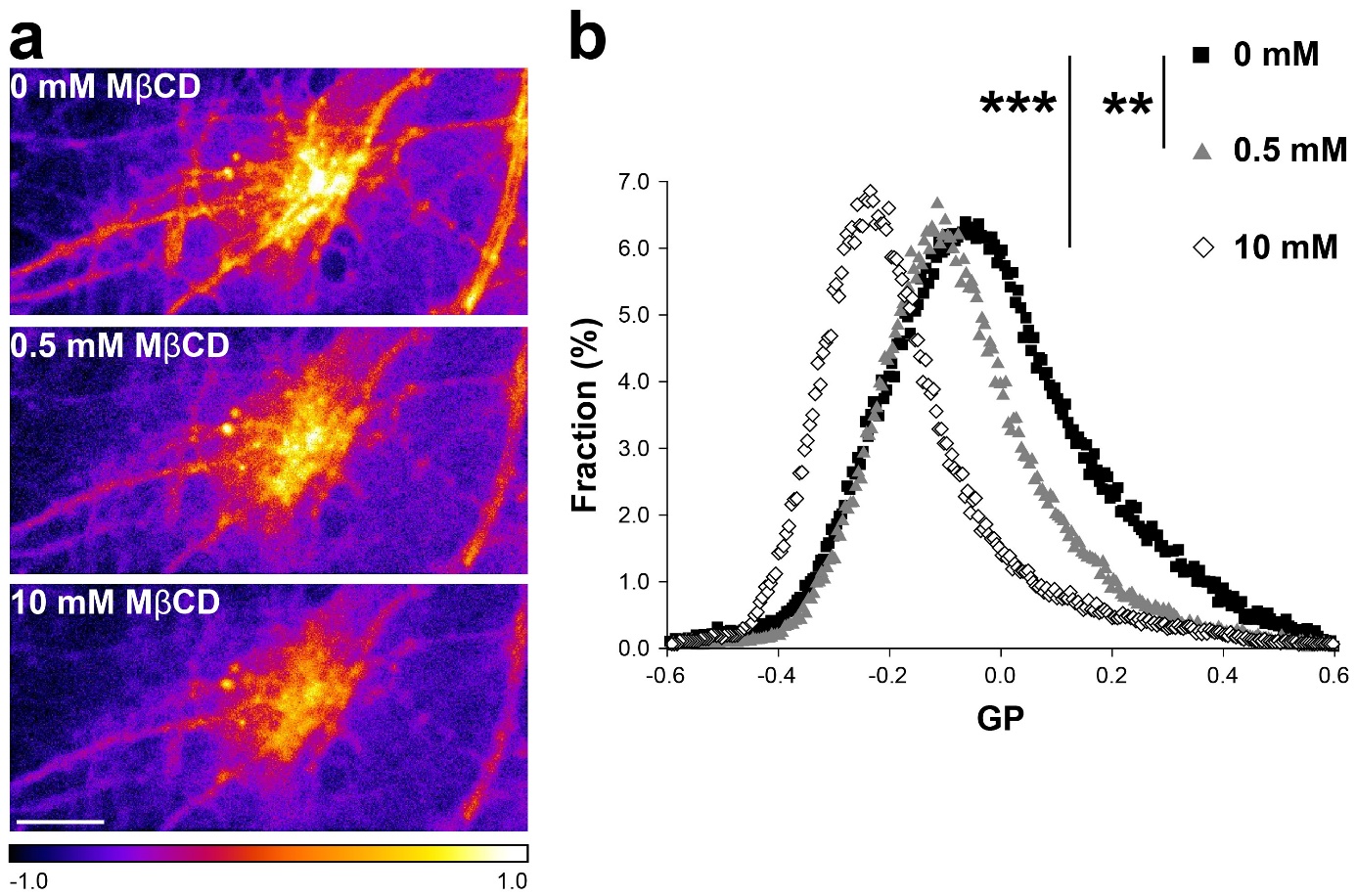
Supplementary Figure 8.** **Cholesterol depletion after MβCD treatment reduces GP values.** (**a**) Sample images of the same field of view after treatment with 0, 0.5-mM 5-min, or 10-mM 30-min MβCD. Scale bar, 20 μm. (**b**) Distributions of GP values over individual image pixels (n = 3 FOVs, N = 3 batches for every treatment; for 0.5 and 10 mM in comparison to 0 mM, *p* < 0.01 and < 0.001 respectively, *Kolmogorov-Smirnov* test of the distributions of GP values, see data analysis section of the methods).**Supplementary Table**

**Supplementary Table 1. Summary Sholl analysis at 12-18 DIV.**

|  | **Graphene** | **Glass** | ***p* - value**  **(one-way ANOVA)** |
| --- | --- | --- | --- |
| **Critical Value** | 78.89 | 98.57 | 0.5567 |
| **Dendrite Maximum** | 17.56 | 20.43 | 0.0721 |
| **Schoenen Ramification Index** | 3.6582 | 5.1643 | 0.0490 |
| **Regression Coefficient** | 0.0439 | 0.0522 | 0.1568 |

**Supplementary Discussion**

**Quantification of the number of quantum dots for total releasable pool vesicles**

For the loading of the total releasable pool of synaptic vesicles, imaging conditions were optimized such that the dynamic range of our data would be covered but not saturated under the loading of Qdots to all recycling synaptic vesicles. Thus the unitary brightness of a single Qdot is relatively low. This setting was optimized on a test coverslip and kept the same for both experimental conditions. Although the brightness of a single Qdot was low above the background, in this set of experiments many Qdots were loaded per synapse, which would thus allow greater certainty in the estimation of the number of total recycling vesicles per synapse. Furthermore, the estimate of the fluorescence of a single Qdot, even with a signal 378 a.u. above background, is easier to quantify given the blinking property Qdots possess: all single quantifications were measured as the difference between a Qdot in its off state and a Qdot in its on state. A more detailed explanation of the use of blinking to help characterize single Qdot photoluminescence can be found in the reference [^2^](#_ENREF_2).

**Supplementary References**

1 Mangan, P. S. & Kapur, J. Factors Underlying Bursting Behavior in a Network of Cultured Hippocampal Neurons Exposed to Zero Magnesium. *Journal of Neurophysiology* **91**, 946-957, doi:10.1152/jn.00547.2003 (2004).

2 Zhang, Q., Li, Y. & Tsien, R. W. The Dynamic Control of Kiss-And-Run and Vesicular Reuse Probed with Single Nanoparticles. *Science* **323**, 1448-1453 (2009).
